# Architecture of Dispatched, a Transmembrane Protein Responsible for Hedgehog Release

**DOI:** 10.3389/fmolb.2021.701826

**Published:** 2021-09-07

**Authors:** Yitian Luo, Guoyue Wan, Xuan Zhou, Qiuwen Wang, Yunbin Zhang, Juan Bao, Yao Cong, Yun Zhao, Dianfan Li

**Affiliations:** ^1^ CAS Center for Excellence in Molecular Cell Science, Shanghai Institute of Biochemistry and Cell Biology, University of Chinese Academy of Sciences, Chinese Academy of Sciences, Shanghai, China; ^2^ School of Life Science and Technology, ShanghaiTech University, Shanghai, China; ^3^ School of Life Science, Hangzhou Institute for Advanced Study, University of Chinese Academy of Sciences, Hangzhou, China

**Keywords:** cryo-EM, hedgehog release, hedgehog signaling, membrane protein, three-dimensional structure

## Abstract

The evolutionarily conserved Hedgehog (Hh) signaling pathway is crucial for programmed cell differentiation and proliferation. Dispatched (Disp) is a 12-transmembrane protein that plays a critical role in the Hedgehog (Hh) signaling pathway by releasing the dually lipidated ligand HhN from the membrane, a prerequisite step to the downstream signaling cascade. In this study, we focus on the Disp from water bear, a primitive animal known as the most indestructible on Earth. Using a zebrafish model, we show that the water bear homolog possesses the function of Disp. We have solved its structure to a 6.5-Å resolution using single-particle cryogenic electron microscopy. Consistent with the evolutional conservation of the pathway, the water bear Disp structure is overall similar to the previously reported structures of the fruit fly and human homologs. Although not revealing much detail at this resolution, the water bear Disp shows a different conformation compared to published structures, suggesting that they represent different functional snapshots.

## Introduction

Multi-cellular organisms rely on proper cell-cell communications mediated by controlled signaling transduction throughout the life cycle. The Hedgehog (Hh) signaling pathway is essential in embryonic morphogenesis in metazoans (Niewiadomski et al., 2019). The malfunction of the pathway results in the clustering of “hairy” denticles in *Drosophila* (fruit fly) larvae, a phenotype that inspired the name of the *hedgehog* gene (Nüsslein-Volhard and Wieschaus, 1980). In humans, abnormal Hh activities are associated with congenital craniofacial deformities (Nieuwenhuis and Hui, 2005) and tumorigenesis (Otsuka et al., 2015; Niewiadomski et al., 2019). In the absence of signals, the pathway is suppressed by a multi-crossing transporter named Patched (Ptc) which depletes sterol in the inner leaflet of the plasma membrane (Myers et al., 2017; Zhang et al., 2018). The Hh ligand released by producing cells binds to Ptc, inhibiting the sterol-transport function and activating the downstream signaling cascade that leads to the transcription of several genes in the pathway (Hui and Angers, 2011).

Hh, the ligand of the pathway, is a family of small proteins sharing similar features. Among them, the Sonic Hh (Shh) is the most studied. After its synthesis in the producing cells, the pre-protein is first cleaved by its own C-terminal catalytic domain. A cholesterol molecule is then attached to the newly exposed C-terminus of the N-terminal half (HhN) (Hall and Cleverdon, 2019), followed by a second lipidation step at its N-terminus by the Hh acyltransferase (Pepinsky et al., 1998). The dual lipidation makes the mature HhN ligands hydrophobic and may serve as a “barcode” for its intracellular trafficking from the endoplasmic reticulum or Golgi to the plasma membrane where Hh is anchored by the lipid moieties (Mao et al., 2009; Kornberg, 2011; Simon et al., 2016). To reach and affect receiving cells in long-range signaling events such as tissue patterning (Etheridge et al., 2010), Hh must be firstly released from the membrane of the producing cells. This process is facilitated by the transmembrane protein Dispatched (Disp) (Burke et al., 1999). Consistent with this important role, malfunction of Disp dispatterns Hh concentration gradient and causes developmental abnormalities in long bones in mice embryo (Tsiairis and McMahon, 2008).

Disp (∼120 kDa) belongs to the resistance-nodulation-division (RND) family (Nikaido, 2018), members of which include the aforementioned Hh receptor Ptc (Qi et al., 2018a; Qi et al., 2018b; Gong et al., 2018; Zhang et al., 2018; Qi et al., 2019; Qian et al., 2019; Rudolf et al., 2019; Zhang et al., 2020; Luo et al., 2021); the cholesterol transporter NPC1 (Gong et al., 2016; Li et al., 2016; Long et al., 2020; Qian et al., 2020); and a variety of bacterial transporters such as AcrB (Seeger et al., 2006). Structural studies have revealed common features of RND transporters (Nikaido, 2018). They typically contain a membrane-embedded domain splitting into two pseudosymmetrical halves with six transmembrane helices (TMHs) each and two extracellular domains that are associated with the two transmembrane halves. RND transporters are known to use electrochemical gradients such as proton- and sodium-motive force as the energy source to drive the conformational cycles (Pos, 2009; Myers et al., 2017). Consistently, three negatively charged residues in the transmembrane domain of Ptc (Asp499, Asp500, and Glu1081 in murine Ptc) are proposed to sense electrochemical gradient and to coordinate the conformational changes (Taipale et al., 2002; Myers et al., 2017; Zhang et al., 2018). Unlike Ptc, of which the transporter function is supported by structural and mutagenesis studies (Myers et al., 2017; Zhang et al., 2018), evidence for Disp being a transporter is somewhat elusive: the cholesterol moiety is required for Disp-mediated Hh release (Tukachinsky et al., 2012), and the genetic neutralization of the corresponding intramembrane negatively charged residues causes loss of function (Ma et al., 2002).

Recently, the structures of Disp from fruit fly (*ff*Disp) (Cannac et al., 2020) and human (*h*Disp) (Chen et al., 2020) were determined by two independent research groups using cryogenic electron microscopy (cryo-EM). The structural studies revealed its architecture with two similarly sized ECDs connected to the 12-TMH domain. In addition, both groups reported the low-resolution map of Disp in complex with the ligand Hh, providing direct evidence for the interaction and revealing the interface information for the binding. Despite the evolutional distance, *ff*Disp and *h*Disp share a superimposable architecture, thus likely representing the same functional conformation snapshot. Obtaining molecular images at different conformations will be helpful to understand its function mechanism. For membrane transporters and channels, this may be achieved by structural studies using conformation-locking mutants (Bozzi et al., 2016; Vuppada et al., 2018; Galochkina et al., 2019), by exploring different membrane environments such as lipid nanodiscs (Gao et al., 2016; Mi et al., 2017; Matthies et al., 2018; Kalienkova et al., 2019; Reddy et al., 2019; Luo et al., 2021), by screening conformation-specific antibodies (Zimmermann et al., 2018; Kumar et al., 2021), and by screening different experimental conditions including protein homologs (Newstead et al., 2011; Solcan et al., 2012; Doki et al., 2013) and buffer conditions (Chang et al., 2014; Qian et al., 2020). In terms of homologs, we had an interest in water bear proteins. Water bears are the most resilient animal known because of their ability (as a collective term for many species) to endure conditions such as exposure to extreme temperatures (−272°C and 150°C), extreme pressures (6,000 atmospheres and vacuum), air deprivation, radiation, dehydration, and starvation (Sloan et al., 2017; Orellana et al., 2018). Despite the emerging interests in using water bear as model systems to study evolutional development (Gabriel et al., 2007; Goldstein, 2018), structural information for their proteins are scarce (Fukuda et al., 2017; Fukuda and Inoue, 2018; Kim et al., 2019) and no membrane protein structures of water bear origin have been reported.

In this study, we report the characterization, expression, purification, and cryo-EM structure determination of Disp from *Hypsibius dujardini*, a type of water bear. The water bear Disp (*wb*Disp) has an overall similar structure with *ff*Disp and *h*Disp, despite that they only share modest sequence homology. Interestingly, the transmembrane domain of *wb*Disp shows a slightly different conformation compared to the published structures. The observations raise the possibility of the structural differences being related to Disp’s function and the structures provide a preliminary framework to design experiments to test this hypothesis.

## Materials and Methods

### Molecular Cloning

The DNA encoding Disp from water bear (NCBI *OQV19566.1*) was obtained by PCR using overlapping oligos that are 60 to 90-nt long. The resulting fragments were Gibson assembled into a pEG_BacMam-backbone vector named pBHGS (Goehring et al., 2014). The polypeptide sequence of protein expressed using this vector contains amino acids of, from N- to C-terminus, as follows: a 9 × His tag, the protein of interest, a 3C protease site, a thermostable green fluorescent protein (Yao et al., 2020), and a twin-Strep tag. Deletions were made by standard PCR-based protocols. DNA sequences were verified by sequencing.

### Protein Expression and Purification

Disp homologs were transiently expressed in Expi293 cells as follows. For small-scale expression, typically, 15 ml of cells (> 95% viability) at a density of 2 × 10^6^ ml^-1^ was diluted by two folds and cultured for another 24 h at 37°C in a 100 ml flask in a CO_2_ incubator to re-reach a cell density of 2 × 10^6^ ml^-1^. Cells were aliquoted to 6-well plates for small-scale expression tests. Plasmid (5 µg) and polyethylenimine (Cat. 23,966–2, Polysciences, 10 µg) were first separately incubated with 200 µL of 293 medium (Cat. UP0050, Union Biotech, China) at room temperature (RT, 18–22°C) for 3 min. The two mixtures were then combined and incubated at RT for 20 min before being added into 2 ml of cell culture. Sodium valproate (Cat. P4543-100G, Sigma) was included as an additive (Wulhfard et al., 2010) to increase expression at a final concentration of 2 mM 12 h after transfection. After 40 h, cells were harvested by centrifugation, flash-frozen in liquid nitrogen, and stored at −80°C until use. For large-scale expression, the same protocol was followed except that 0.5 L of cells was seeded to 1 L of culture and the transfection was performed with 2.5 mg of plasmids premixed with 5 mg of polyethylenimine.

For purification, biomass from 2 L of culture was re-suspended lysis buffer (0.5 mM EDTA, 20 mM HEPES pH7.5 and 150 mM NaCl) supplemented with 2 mg ml^-1^ iodoacetamide, complete protease inhibitor cocktail (Cat. 11836153001, Roche). Solubilization was carried out by adding 1% (w/v) *n*-dodecyl β-D-maltoside (DDM) plus 0.2% (w/v) cholesteryl hemisuccinate (CHS) to the cells with mild agitation at 4°C for 20 min. Cell debris was removed by centrifugation at 48,000 g for 1 h. The supernatant was mixed with Strep Tactin resin (Cat. 2–1201–010, IBA) that had been pre-equilibrated with the Buffer A and mixed gently for 2 h. After batch binding, the beads were loaded to a gravity column and washed by 15 column volume (CV) of 0.1% digitonin in Buffer A. *wb*Disp-GFP was then eluted from the column by 5 mM of dethiobiotin and 0.1% digitonin in Buffer A. The eluted *wb*Disp-GFP fractions were quantified by absorbance at 493 nm (ε_493_ = 63,973 M^-1^ cm^-1^) and the fluorescent tag was removed by 3C protease cleavage at 4°C overnight. The mixture was then centrifuged at 21,000 g for 10 min before being loaded onto a Superose 6 10/300 GL column pre-equilibrated by Buffer A containing 0.1% digitonin for gel filtration. Peak fractions were pooled and concentrated to 3 mg ml^-1^.

### Fluorescence-Detection Size Exclusion Chromatography (FSEC) Assay for Expression and Thermostability

FSEC assays (Hattori et al., 2012; Yao et al., 2020) were carried out to assess expression level and apparent thermostability as follows. Cells expressing Disp homologs or variants with a thermostable GFP (TGP) tag were re-suspended in 200 µL of FSEC Buffer (0.2 mM tris(2-carboxyethyl)phosphine (TCEP), 50 mM NaCl, and 20 mM Tris-HCl pH 8.0, 0.5 mM EDTA) supplemented with 1 × protease inhibitor cocktail. DDM and CHS were added to a final concentration of 1% (w/v) of DDM and 0.2% (w/v) of CHS for solubilization at 4°C for 20 min. The solubilized fraction was clarification by centrifugation at 21,000 g for 30 min before FSEC assays below.

To assess their relative expression level, 2 µL of solubilized materials was loaded onto a ZenixC-300 column in a Shimadzu or Agilent HPLC machine equipped with a fluorescence detector for signal monitoring at the 482/508 nm pair. The mobile phase contained 0.03% DDM (w/v) and 0.006% CHS (w/v), 0.2 mM TCEP, 50 mM NaCl, 0.5 mM EDTA, and 20 mM Tris-HCl pH 8.0. The intensity and profile of the FSEC peak at desired V_e_ were compared between Disp homologs, Disp mutants, or expression conditions such as time post-transfection, temperature, and types (sodium valproate and sodium butyrate) and concentration of additives.

For FSEC-based thermostability assays, 50 μL of cell lysate was heated in a PCR machine for 20 min, followed by 4°C incubation for 10 min. The heated samples were clarified by centrifugation before being loaded onto a ZenixC-300 column in a Shimadzu or Agilent HPLC machine equipped with a fluorescence detector for signal monitoring at the 482/508 nm pair. The % fluorescence of the Disp-TGP peak was plotted against temperature and the apparent melting temperature (*T*_m_) was obtained by regression fitting.

### Zebrafish Rescue Assay

Zebrafish (*Danio rerio*) of the AB strain was provided by the Zebrafish Core Facility at the authors’ institute, and all experimental protocols were approved by the Institutional Animal Care and Use Committee. sgRNAs were designed using the CRISPR Design webserver CCTop (https://crispr.cos.uni-heidelberg.de/index.html) and/or CHOPCHOP (http://chopchop.cbu.uib.no/). DNA templates for sgRNAs were PCR-amplified using the following forward primers containing a T7 promoter (italic) and guide sequence (Disp11, 5’- *TAA​TAC​GAC​TCA​CTA​TA*GGT​GTC​CCA​GCA​TTC​AGG​ACC​GGT​TTT​AGA​GCT​AGA​AAT​AGC-3’; Disp12, 5’- *TAA​TAC​GAC​TCA​CTA​TA*GGA​TGG​GAT​CAC​GAC​TAT​AAG​TTT​TAG​AGC​TAG​AAA​TAG​C-3’; Disp13, 5’- *TAA​TAC​GAC​TCA​CTA​TA*GGA​GTT​GAA​GAC​CAC​TCG​ATA​TGT​TTT​AGA​GCT​AGA​AAT​AGC-3’) and the reverse primer with the standard chimeric sgRNA scaffold (ScaRev, 5’-AAA​AGC​ACC​GAC​TCG​GTG​CCA​CTT​TTT​CAA​GTT​GAT​AAC​GGA​CTA​GCC​TTA​TTT​TAA​CTT​GCT​ATT​TCT​AGC​TCT​AAA​AC-3’) (Varshney et al., 2015). sgRNAs commentary to the PCR products was synthesized *in vitro* using the HiScribe T7 Quick High Yield RNA Synthesis Kit (New England Biolabs, Cat. E2050S). To generate DNA temperate for the mRNA of *wb*Disp, PCR was performed with the primer pair *wb*D-Fwd (5’- *TAA​TAC​GAC​TCA​CTA​TA*GGA​TGC​CGA​GAC​AAC​TCG​TGC​GAC​GGC-3’) and *wb*D-Rev (5’- TCA​CTC​GGT​CGT​CCG​TCG​CAC​CC-3’) using the pBHGS-*wb*Disp plasmid as the template (in the primer sequence, italic denotes sequence representing a T7 promoter). *In vitro* synthesis of mRNA containing a 5’-cap and a 3’-poly(A) tail was performed using the mMessage mMachine SP6 kit (Ambion, Cat. AM1340). Cas9 ribonucleoprotein (RNP) complexes were prepared with Cas9 protein and sgRNAs as previously described (Varshney et al., 2015). The RNPs were injected into one-cell stage zebrafish embryos with or without mRNAs. Each embryo was injected with 1 nL of a solution containing 5 μM Cas9 and 1 μg L^-1^ sgRNA or ∼5 μM Cas9, 1 μg μL^-1^ sgRNA, and 160 ng μL^-1^ mRNA. 24 hours after fertilization (hpf), larvae were fixed in 4% paraformaldehyde (PFA; w/v, pH 7.4) overnight at 4°C, washed with PBS, and fluorescently stained with 4’,6-diamidino-2-phenylindole (DAPI). All specimens were washed with PBS before imaging under an Olympus FV3000 confocal laser scanning microscopes.

### Cryo-EM Sample Preparation and Data Acquisition

Two microliters of protein was applied to a Quantifoil Cu R1.2/1.3 (200 mesh) grid that was glow-discharged followed by blotting using Vitrobot Mark IV (FEI company) and flash-vitrified in liquid ethane. Movie stacks were acquired using a 300-kV Titan Krios transmission electron microscope (Thermo Fisher) equipped with a Cs corrector onto a K2 Summit direct electron detector (Gatan) in super-resolution mode. The pixel size was 1.0 Å after 2 times of binning (Supplementary Table S1). Each movie was dose-fractioned into 38 frames using a dose rate of 8 e^-^ per physical pixel per second on the detector. The exposure time was 7.6 s (0.2 s for each frame) with a total dose of 60.8 e^-^/Å^2^. Defocus values varied from −1.5 to −2.5 μm. All the images were collected with the SerialEM automated data collection software package (Mastronarde, 2005).

### Cryo-EM Data Processing

Data processing was carried out using modules either belonging to or available through Relion 3.0 and 3.1 (Supplementary Figure S2) (Scheres, 2012a; Scheres, 2012b). Two datasets containing 2,852 and 2,528 movie stacks were motion-corrected and dose-weighted before further processing. Parameters of the contrast transfer function (CTF) were estimated using Ctffind 4 (Mindell and Grigorieff, 2003) and micrographs with CTF fitting quality better than 4 Å were used for particle extraction. To construct a template for automatic particle picking, we manually selected ∼2,000 particles for reference-free 2D classification. A total of 433,048 particles from dataset one and 345,698 particles from dataset two were obtained after removing bad particles. The particles were twofold downsampled to accelerate processing and subjected to several rounds of 2D classification, resulting in 184,167 and 182,925 clean-up particles from each subset. Subsequently, 3D classification was performed on each set using particles from selected 2D classes. After adjusting parameters (class number K, regularization parameter T, and particle alignment parameters), 3D classes with clear structural features were selected from each dataset. This resulted in 84,596 particles in Dataset 1 and 69,183 particles in Dataset 2. Further rounds of 3D classification using combined particles from the two datasets yielded a class with 62,850 particles. These particles were refined with a protein-only mask and further applied to CTF refinement and Bayesian polishing. Polished particles were refined with the mask and sharpened into a 6.5 Å map by post-processing. Although the second refinement step did not improve the overall resolution, it yielded a map displaying more features in TMHs. Local resolution was estimated using Relion (Scheres, 2012b).

### Model Building and Validation

Model of the *ff*Disp structure (PDB ID 6tbu) trimmed to Cα was fit to the 6.5-Å map using UCSF Chimera (Pettersen et al., 2004). The model was then manually adjusted based on the cryo-EM density map using Coot (Emsley et al., 2010) and refined in real space using Phenix (Liebschner et al., 2019). The resulting model was finely adjusted to fix geometry violations guided by the cryo-EM density map using Coot.

## Results and Discussion

### *wb*Disp Shows a Higher Expression Level Than Other Homologs

**FIGURE 1:**
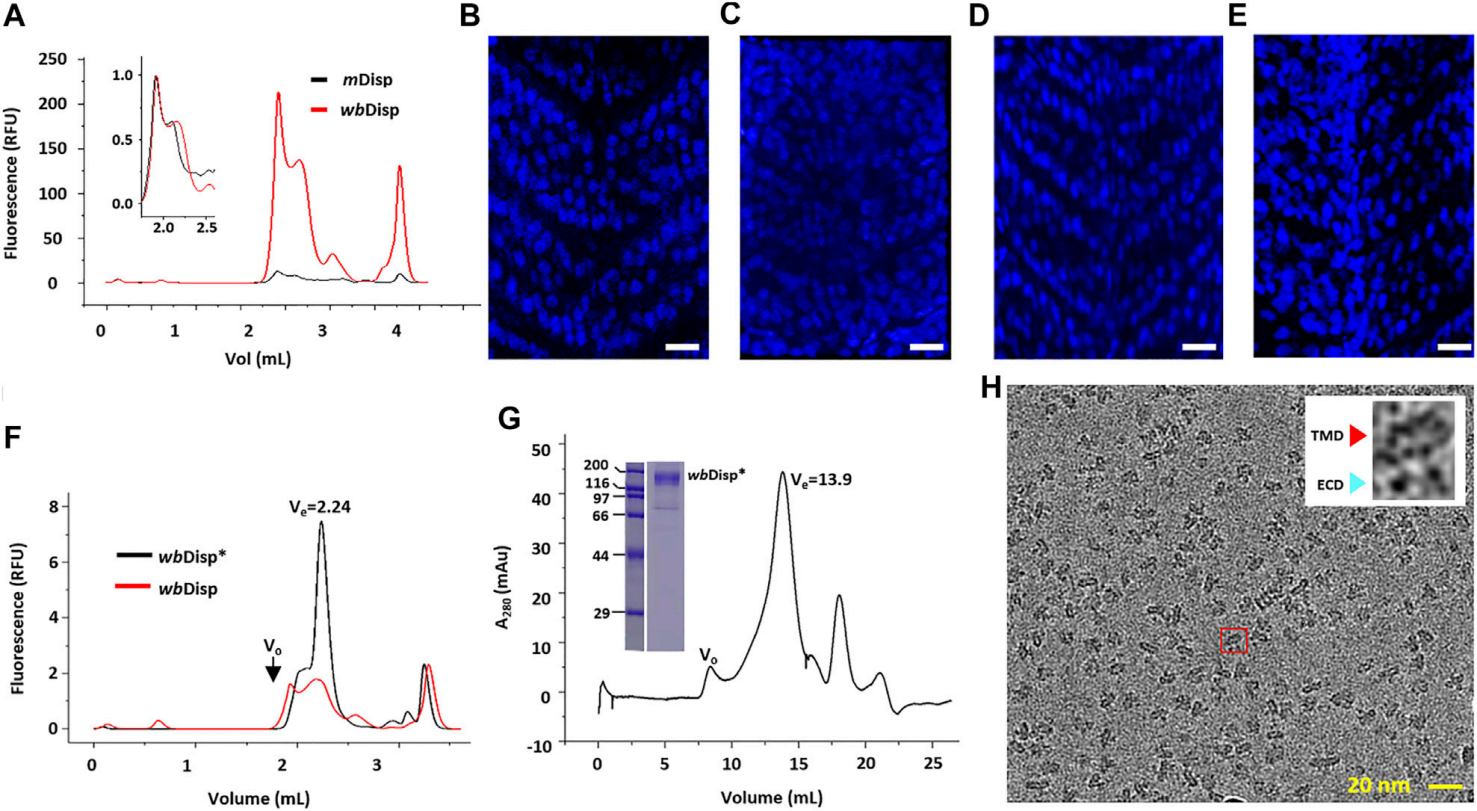
Screening Disp homologs for structural and functional studies. **(A)** Fluorescence-detection size exclusion chromatography (FSEC) of Disp from mouse (*m*Disp) and water bear (*wb*Disp). The void volume was 1.80 ml and the total volume was 4.20 ml. **(B–E)** Phenotype-rescue assay of *wb*Disp. The V-shaped somites seen in zebrafish **(B)** were lost upon the knockout of the endogenous *disp* gene **(C)**. This phenotype could be fully or partly rescued by introducing *m*Disp **(D)** or *wb*Disp mRNA to the embryo **(E)**. Bar = 40 μm. The brightness and contrast of **(C)** were adjusted as a whole for better contrast. **(F)** FSEC of the wild-type *wb*Disp and *wb*Disp* which contained several deletions (see text). **(G)** Preparative gel filtration of *wb*Disp. The void volume (V_0_) is appropriately labeled. Inset shows the SDS-PAGE of a main-peak fraction. Molecular standards for electrophoresis are shown as kDa on the left. **(H)** A typical cryo-EM micrograph of *wb*Disp purified in digitonin. Inset shows the expanded image of a particle (red box) with a typical side view displaying a transmembrane domain (TMD) and two extracellular domains (ECDs).

To elucidate the then-unknown architecture of Disp, we first set to overexpress, purify, and determine the cryo-EM structure of the murine homolog (*m*Disp) as it is one of the most studied in the literature. *m*Disp was expressed in Expi293 cells with a thermostable green fluorescence protein (TGP) fused to its C-terminal (Cai et al., 2020). This allows convenient assessment of gel filtration behavior and expression level without purification using fluorescence-detector size exclusion chromatography (FSEC). As shown in Figure 1A, multiple peaks were observed for *m*Disp due to degradation and aggregation. The main peak had a V_e_ of 2.24 ml which was similar to the V_e_ of the monomeric (Luo et al., 2021) Ptc1 (2.40 ml), a homolog of Disp with a similar architecture. Thus, *m*Disp likely formed a monomer under current conditions.

Preliminary purification of *m*Disp using Strep resin only yielded ∼50 µg of protein with insufficient purity (∼70%) from 1 L of *m*Disp-overexpressing mammalian cells. This relatively low yield prompted us to screen homologs for higher expression levels by FSEC analysis (Cai et al., 2020). Thus, six additional homologs were expressed in mammal cells on small scales, and the relative yields reflected by the peak intensities were assessed by FSEC using detergent-solubilized lysates. Among seven homologs (water bear, sea urchin, coral, slug, scorpion, pupfish, and mouse) (Supplementary Figure S2), *wb*Disp showed about 20-fold higher expression than *m*Disp. When superposed, *wb*Disp showed a similar FSEC profile to *m*Disp (Figure 1A).

### *wb*Disp Partially Rescues Somite Structure in *disp* Knock-out Zebrafish

To investigate the function of *wb*Disp, a phenotype-rescue assay was performed as follows to see if *wb*Disp mediates Hh signaling in zebrafish. We injected mRNA of *wb*Dsip and *m*Disp into the embryo of zebrafish where the endogenous *disp* was knocked out using CRISPR/Cas9. In the absence of *disp*, the somite structure of zebrafish transformed from a V-shape (Figure 1B) to a U-shape (Figure 1C). Injecting mRNA of *m*Disp into the embryo completely rescued the phenotype (Figure 1D) while injecting *wb*Dsip mRNA resulted in partial rescue (Figure 1E), suggesting that *wb*Disp can partially recover Disp’s activity. It may be that the non-native lipid environment and non-native HhN ligand (sequence identity of 59.6%) (Supplementary Figure S3) were not optimal for the heterogeneously expressed *wb*Dsip, resulting in an incomplete rescue.

### Purification and Negative Staining of *wb*Disp

To further optimize the expression level, we screened several *wb*Disp constructs by removing regions that are predicted (McGuffin et al., 2000) to be flexible (Supplementary Figure S4). First, the C-terminus (residues 986–1192, inclusive) was systematically truncated by 50–70 residues at a time. The construct containing residues 1–1116 was further screened by deletion at the internal loops. Specifically, the regions spanning residues 80–160, 530–550, and 660–680 were firstly systematically tested by removing approximately ten residues at a time. The ones with higher yields were then combined. This yielded the construct (dubbed *wb*Disp*) that consists of residues (1–111, 123–530, 542–1116) (Supplementary Figure S4). The optimized construct showed a three-fold higher expression level compared to the wild type (Figure 1F). To assess its stability, we performed the FSEC-thermostability assay (Hattori et al., 2012; Cai et al., 2020) of *wb*Disp using the tagged TGP as the reporter. Thus, the samples were heated for 20 min under different temperatures and the fluorescence intensity of the FSEC peak was plotted against temperature. As shown in Supplementary Figure S5A, *wb*Disp* displayed an apparent *T*_m_ of 79°C. Compared to other membrane proteins (Cai et al., 2020), the denaturation profile shows a relatively slow decrease in the descending phase. This may indicate reversible denaturation.

To purify *wb*Disp, the protein was solubilized in DDM/CHS (1% DDM and 0.2% CHS) and the detergent was exchanged to 0.1% digitonin on a Strep-Tactin column. On gel filtration, *wb*Disp* showed a reasonably symmetrical main peak. When analyzed by SDS-PAGE, the peak fraction contained mainly the target protein (Figure 1G). Negative-stain of purified *wb*Disp* showed well-dispersed particles with views consistent with a membrane protein with two extracellular loops (Supplementary Figure S5B).

### Cryo-EM Structure of wbDisp*

**FIGURE 2:**
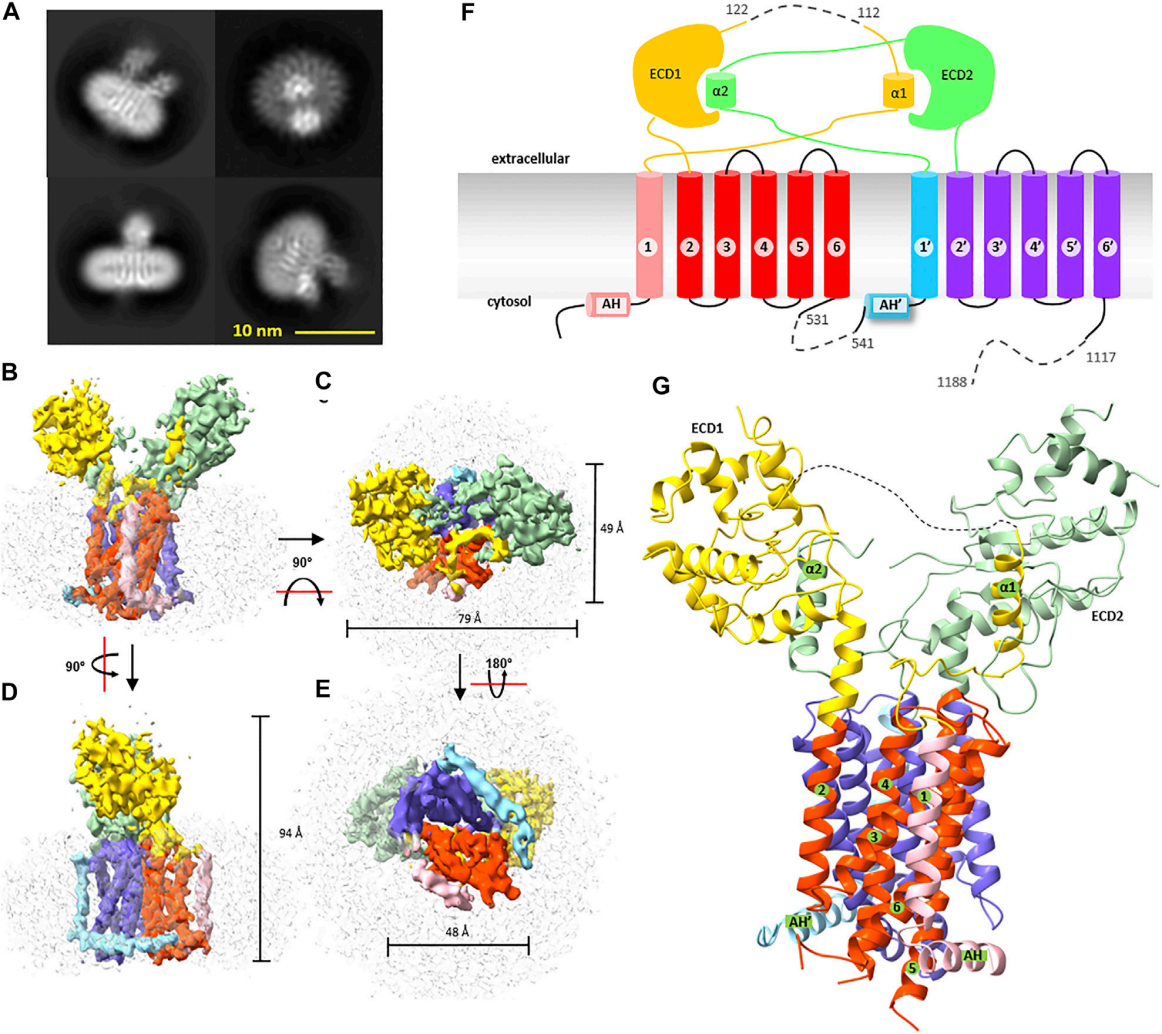
Cryogenic electron microscopy structure of *wb*Disp. **(A)** Typical 2D class average of the cryo-EM particles. **(B–E)** Different views of the 3D map reconstructed from 62,850 particles. The density of the micelles is colored grey. Various parts of the protein are colored as in **(F)** and **(G)**. **(F)** Membrane topology of *wb*Disp. Numbers with and without prime symbols indicate transmembrane helices in the N-terminal half and C-terminal half, respectively. ECD: extracellular domain; AH: amphipathic helix. Dashed lines denote deletion. **(G)** The overall structure of *wb*Disp. Various parts as in **(F)** are labeled, when appropriate.

To determine its 3D structure, *wb*Disp* purified in digitonin was subjected to cryo-EM analysis. A 6.5-Å map was reconstructed using 62,850 particles (Figure 1H, Figures 2A–E, Supplementary Figure S6–S8), showing a slingshot shape with the two extracellular blobs as the branches and the transmembrane helices as the handle (Figure 2B). To obtain a model at this low resolution, the recently published structure of the fruit fly homolog (*ff*Disp) (Cannac et al., 2020) was trimmed to poly-alanine and fit into the map, followed by manual mutation and adjustments.

*WB*Disp showed a monomeric structure with 12 transmembrane helices (Figures 2F,G). In literature, trimeric assembly by C-terminal regions has been reported for the dog Disp (Etheridge et al., 2010). Because our constructs were truncated at the C-terminal region, it is currently unknown if the wild type also exists as a trimer. However, since the wild-type *wb*Disp had a similar retention volume as the truncated *wb*Disp* (Figure 1F) on size exclusion chromatography, the protein engineering probably did not change the oligomerization state, at least under the micellar conditions. The possibility of Disp as oligomers remained to be investigated.

The *wb*Disp structure has a dimension size of approximately 49, 79, and 94 Å. When viewed from the cytosol, the transmembrane region is square-shaped with a dimension of 49 by 48 Å (Figures 2B–E). The two large extracellular domains (ECDs) occupy a similar volume of ∼ 3,350 Å^3^.

The transmembrane domain shares a similar fold to other RND members. It contains two pseudosymmetrical repeats (Figure 2F, Supplementary Figure S9) with each repeat containing six TMHs and a soluble domain. Proceeding to the 6-TMH bundle is an amphipathic helix that lies on the membrane interface at the cytosol leaflet.

**FIGURE 3:**
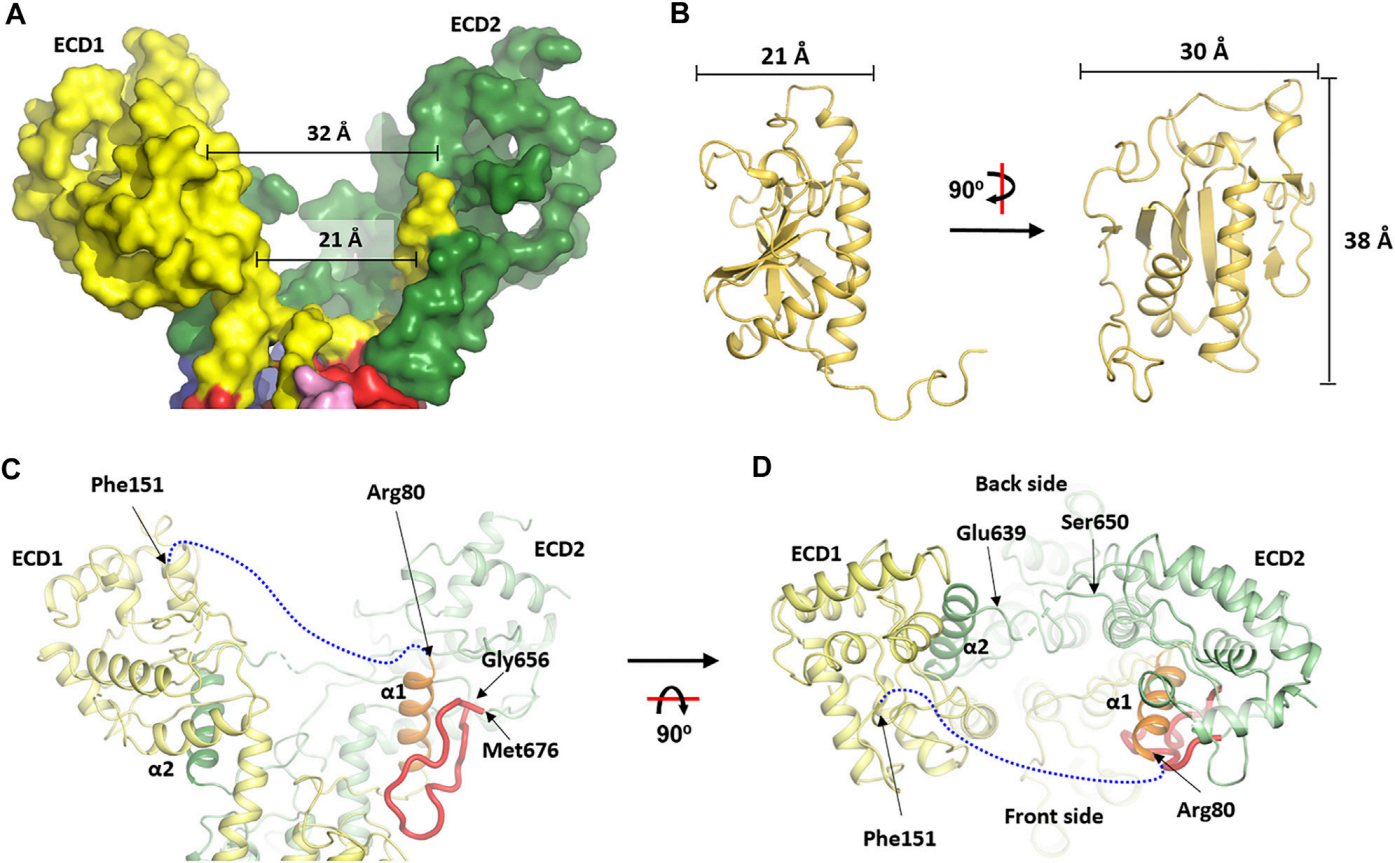
The asymmetry of the extracellular domain and the conformational changes at the transmembrane domain. The two interconnected extracellular domains (ECDs) form the two arms of a slingshot and creates a cavity that is more open at the “front” side than at the “back” side. **(A)** surface view of the ECD part. **(B)** Dimension of the human HhN (PDB: 3M1N) (Pepinsky et al., 2000) which is close to the size of the cavity in **(A)**. **(C, D)** Cartoon representation of the *wb*Disp structure at the two ECDs viewed from two different angles. Various parts are labeled when appropriate. A blue dash line indicates the ECD-connecting loop that was invisible in the current structure.

The two slingshot braches create a wide opening with a gap of ∼21 Å at the bottom and of ∼32 Å at the top (Figure 3A), enough to accommodate an HhN molecule which has a dimension of ∼20 × 30 × 38 Å (Figure 3B). The opening is accessible from the vast extracellular space. Interestingly, when viewed perpendicular to the membrane, the exit to the “back” side (we name the site above TMH1-6 as the “front” side and the opposite as the “back” side) is somewhat restricted by a loop (res. 640–648) that connects the two branches (Figures 3C,D). Thus, embracing HhN in this direction would require large conformational rearrangements. By contrast, the loop (res. 80–159) at the “front” side is much longer and highly flexible (invisible in the structure), suggesting a more accommodating entrance for the presumable lateral diffusion of the membrane-anchored HhN.

One unique feature of the soluble domain is the way the two loops are interconnected. Thus, a small α-helix (α1, res. 66–79) stemming from TMH1 snakes to the slingshot branch made mostly by the ECD2 residues, before reaching back to form the other branch (ECD1) (Figures 3C,D). Reciprocally, the corresponding α-helix in the C-half (α2, res. 623–639) stemming from TMH7 also travels to the ECD1 part before making ECD2 (Figures 3C,D). Interestingly, α1 is clamped tightly onto the core ECD2 domain by a long loop while α2 only interacts with the core ECD1 at one side (Figures 3C,D). It might be that the binding and release of Hh is influenced by the formation and collapse of these unusual structural arrangements which may mediate cross-talk between the two ECDs.

### Structural Differences in the Transmembrane Domain and Possible Functional Consequences

**FIGURE 4:**
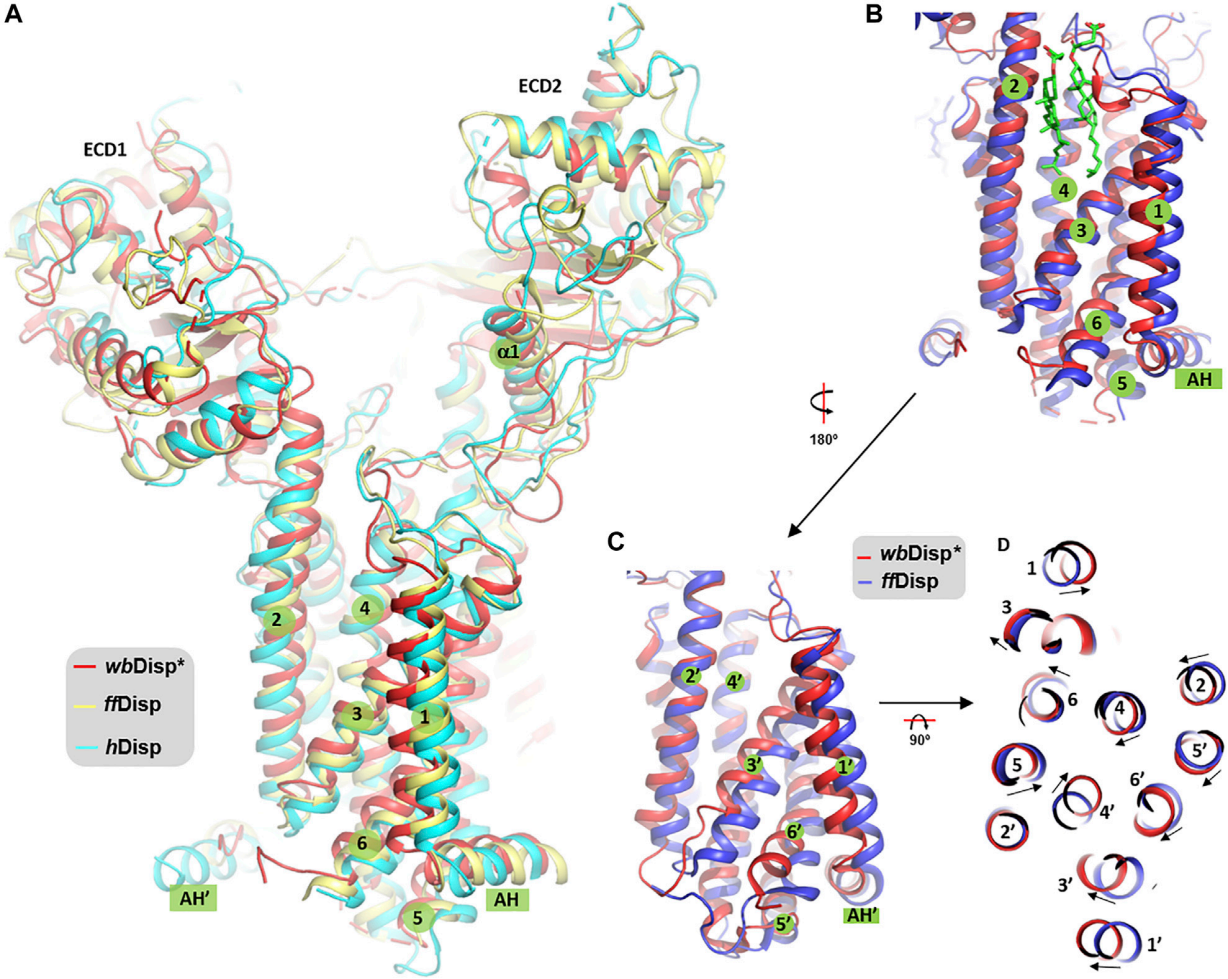
Comparison of Disp structures. **(A)** The overall view. **(B, C)** Comparison of TMH 1–6 **(B)** and TMH 7–12 (TMH1’-6’) **(C)** of *wb*Disp (red) with the *ff*Disp structure (blue). **(D)** View from the extracellular side. The two sterol molecules in **(B)** are from the *ff*Disp structure (PDB ID 6tbu) (Cannac et al., 2020). AH: amphipathic helix.

Recently, structures of the Disp from fruit fly (*ff*Disp) (Cannac et al., 2020) and human (*h*Disp) (Chen et al., 2020) have been reported. In addition to the apo form, the *ff*Disp structure was also determined in complex with the ligand HhN. Interestingly, these published structures display little differences (Figure 4A). By contrast, noticeable differences, particularly at the transmembrane domain, were observed between the *wb*Disp* and the published structures (Figures 4B–D). Specifically, the inner leaflet part of TMH1, TMH3, and TMH6 shift towards TMH2, compressing the membrane core region (Figure 4D), and this is repeated for the TMH1’, TMH3’, TMH6’, and TMH2’ in the C-terminal pseudosymmetrical half.

Although we acknowledge that the structural differences observed here should be treated with caution and should only be interpreted to a confident level the low-resolution map would allow, the consequences of the structural shift are discussed below. The cleft between TMH2 and TMH3 is a sterol binding site, as revealed by the structure of *ff*Disp (Figure 4B); and this site, in the so-called sterol-sensing domain (SSD), is part of a sterol-transporting conduit for the homologous Ptc1 (Kowatsch et al., 2019) which consists of at least three sites (*S1-S3*, Supplementary Figure S10A). Additionally, in Ptc1, this conduit can be “plugged” by the N-terminal palmitoyl chain of Hh at the immediate downstream site *S2* (Supplementary Figure S10B), that is, the Hh-binding activity and the sterol-transport activity are structurally antagonistic. It is therefore possible that the conformational changes at this region are related to the sterol-transport function of Ptc1, and by extension, the function of Disp assuming the two share a similar transporting mechanism. From the current limited resolution, it is not clear whether and how the conformational changes facilitate the binding and release of Hh. Future structural studies in more native-like lipid environments at higher resolutions are warranted to test and amend this hypothesis.

## Data Availability

The cryo-EM map has been deposited to EMDB with accession code EMD-31595 and the structure coordinates are available through the protein data bank (PDB) with entry ID 7FIF.
