# Supplementary material for: Architecture of Dispatched, a Transmembrane Protein Responsible for Hedgehog Release
Source: Front Mol Biosci. 2021 Sep 7;8:701826. doi: 10.3389/fmolb.2021.701826 (PMC8453165; doi:10.3389/fmolb.2021.701826)
Supplement: Supplementary file 1 [file DataSheet1.pdf]

Architecture of Dispatched, a Transmembrane Protein Responsible for Hedgehog Release

Authors: Yitian Luo[1,2,#], Guoyue Wan[1,#], Xuan Zhou[1,#], Qiuwen Wang[1,2], Yunbin Zhang[1], Juan Bao[1], Yao Cong[1], Yun Zhao[3,*], Dianfan Li[1,*]

Affiliations:
[1]CAS Center for Excellence in Molecular Cell Science, Shanghai Institute of Biochemistry and Cell Biology, University of Chinese Academy of Sciences, Chinese Academy of Sciences, 320 Yueyang Road, Shanghai 200031, China.

[2]School of Life Science and Technology, ShanghaiTech University, 393 Middle Huaxia Raod, Shanghai 201210, China.

[3]School of Life Science, Hangzhou Institute for Advanced Study, University of Chinese Academy of Sciences, Hangzhou 310024, China

[#]Equal contribution.

***Correspondence:** **dianfan.li@sibcb.ac.cn**; **yunzhao@sibcb.ac.cn**

Supplementary Information

- Supplementary Figure 1-10
- Supplementary Table 1
- Supplementary References 1-16

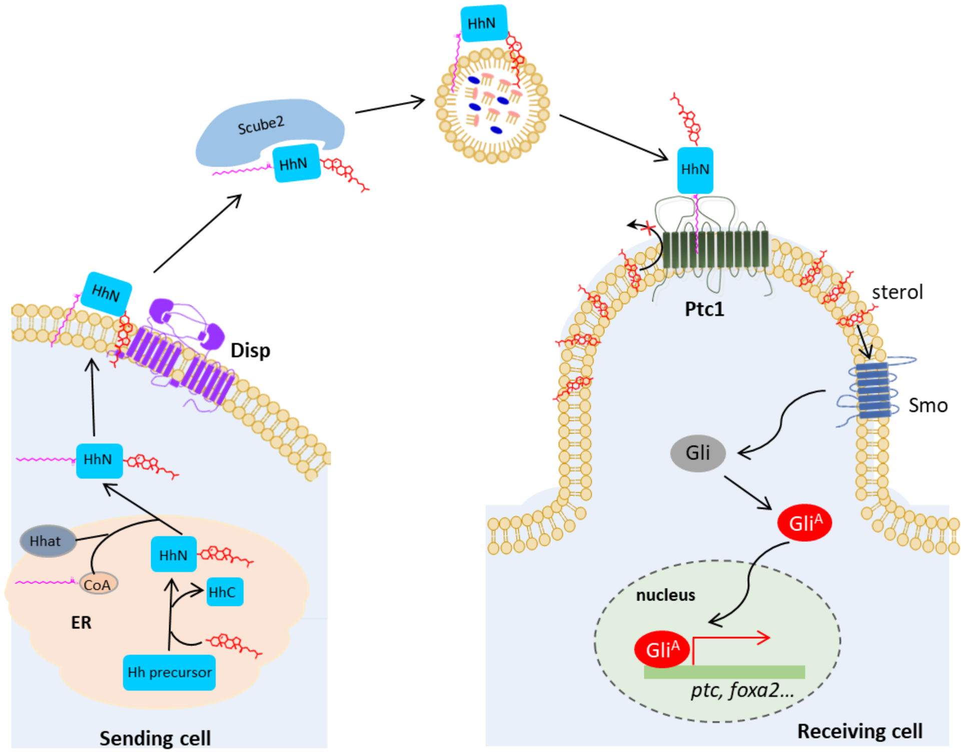

Supplementary Fig. 1. Schematic of the Hedgehog signaling pathway. The Hh precursor is autocleaved and cholesteryl-modified by its C-terminal catalytic domain, before being further lipidated by the acyltransferase Hhat at the N-termius to generate the mature, dually lipidated HhN (1-3). The lipidation enables its intracellular transport from the endoplasmic reticulum (ER) or Golgi to the plasma membrane, a process that is reported to involve caveolin-1 (4). The membrane-tethered HhN is then released by Disp, a process that also requires the co-receptors BOC and CDON (5) before being handed to the secreted protein Scube2 (6, 7). The complex, or self-oligomers as reported in some studies (8) (not drawn here), enters exovesicles or lipoprotein particle (9-11) *via* unknown mechanisms for intercellular trafficking towards receiver cells where it binds and inhibits Ptc1 which functions as a negative regulator for Smoothened (Smo) by depleting cholesterol from the inner membrane leaflet (12). The HhN binding inhibits the cholesterol transport and therefore activates Smo which in turn activates the Gli-mediated cascade that includes the transcription of several Hh-driven genes such as *ptc* and *foxa2* (13).

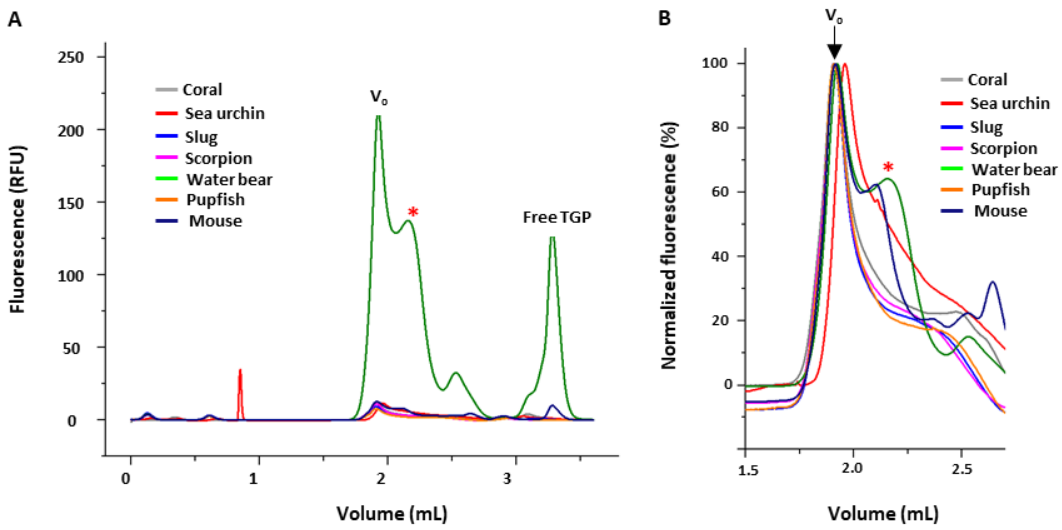

Supplementary Fig. 2. The water bear Disp shows the highest expression level among seven homologs. The Disp homologs were C-terminally tagged with a thermostable green fluorescence protein (TGP) for fluorescence-detection size exclusion chromatography (FSEC). (**A**) FSEC of the seven homologs. Cell lysates were analyzed. The relative fluorescence unit (RFU) was plotted and peak intensity serves as an indication of relative expression level. (**B**) Comparison of the peak profile by normalizing the FSEC trace in **A**. The void volume (V_0) and the desired peak (*) are labeled. The NCBI accession codes for the homologs tested are as follows: coral (*Stylophora pistillata*), XP_022787142.1; sea urchin (*Strongylocentrotus purpuratus*), XP_030854402.1; slug (*Aplysia californica*), XP_005097030.2; scorpion (*Centruroides sculpturatus*), water bear (*Hypsibius dujardini*), OQV19566.1; XP_023223108.1; pupfish (*Cyprinodon variegatus*), XP_015260812.1, and mouse (*Mus musculus*), AAN64660.1.

A

```
zfShh           ---------- --MRLLTRVL LVSLLTLSLV VSGLACGPGR GYGRRRHPKK LT---PLAYK QFIPNVAEKT LGASGRYEGK ITRNSERFKE
wbHh       1    MKLLLPELRS PRRLSLLHLV FFCVLMMRFV TSCDSCGPGR PGMKRTGPVK KYHNKPLREK QFLPNVDEFS GKASGPPEGK ILRKTRRFHD
                             * ::: :..:* : :* .*  :*****    :*  * *      **  * **:*** * :    ***  *** * *::.**::

zfShh      76   LTP---NYNP DIIFKDEENT GADRLMTQRC KDKLNSLAIS VMNHWPGVKL RVTEGWDEDG HHFEESLHYE GRAVDITTSD RDKSKYGTLS
wbHh       91   HMVRVSERNR NIIFRDEERT GADRFMTRRC KEKLEILAIM VRNEWPKVKL FVTEAFDENY EHRKQSLHYE GRALDVKTSD RDRKKYGMLA
                      : *  :***:***.* ****:**:** *:**: *** * *.** ***  ***.:**:  .* ::***** ***:*:.*** **:.*** *:

zfShh      163  RLAVEAGFDW VYYESKAHIH CSVKAEN--- SVAAKSGGCF PGSALVSLQD GGQK------ -AVKDLNPGD KVLAAD-SAG NLVFSDFIMF
wbHh       181  RLAVQAGFDW VFFQSKASIH VSCKSEKGEF EEHLKDERCF HAHNSSVLRK TSPDSNTTET IPMTDLRIGD HVLSTDPSSG TPIFSEMIAW
                ****:***** *:::*** **  * *:*:     .    *.   **  .      *:.  . .        .:.**. ** :**::* *:* . :**::* :

zfShh      242  TDRDSTTRRV FYVIETQEPV EKITLTAAHL LFVLDNSTED LHTMTAAYAS SVRAGQKVMV VDDS------ ---GQLKSVI VQRIYTEEQR
wbHh       271  IDSDLTSQSA FLRVHTAN-G RHISLTPSHL VHVFDVSTR- --AMTTRYAR DLSPGDSLMA LDNANEASIR NPFSTLTAEK IVRIEPVVAV
                 * * *:: . *  :.* :   .:*:**.:** :.*:* **.     :**: **  .: .*:.:*.  :*::          . *.:    : ** .

zfShh      322  GSFAPVTAHG TIVVDRILAS CYAVIEDQGL AHLAFAPARL YYYVS----- ---------- ---------- ---------- ----------
wbHh       357  GSLAPLTRQG TIVVNGISAS CYAAVADHKM AHAAFAPFRW WTLLFGVGRS PQPPDDGMHP NMASRTEGVF TLTDLPLMCI VYPVFRAKCA
                **:**:* :* ****: * ** ***.: *: : ** **** *  :  :

zfShh      368  ---------- ---------- SFLFPQNSSS RSNATLQQEG VHWYSRLLYQ MG-------- ---------- ---------- -TWLLDSNML
wbHh       447  VSEATEHFRA EDREALGKEL IRLEEESSDF LSNSMIEEKN IKEEMRLLDI LLGRFRAAKS DDQERLIIAT MRKLIEKKNL VTELYKNKAA
                                           *  :.*.   **: ::::. ::   ***     :                               * * ..:

zfShh      409  HPLGMSVNSS ---------- ---------- ---------- ---------- ---------- ---------- ---------- ---
wbHh       537  NDTRGTQNSN GQASGTNGDP EGTRSPAGRQ SEGPAGVGAC PVVYVVQRNC NNGEEEGDSD ESSNEIRGDG RSQDQLKATQ SGE
                :    : **.
```

B

```
wbDisp          ---------- ---------- ---------- ---------- ---------- ---------- ---------- ---------- ----------
zfDisp     1    MALSEDFTEL QTLSNGRSSS IFPTTNTPVD PEEEEEQSVS ACTQPNEHTQ MMQTGQNGGL KSTRLSSSSS SSSQLTDAQR LAVLLPNALP

wbDisp          ---------- ---------- ---------- ---------- --------MP RQ-------- -LVRRRKKTV MQRYAHFLAH HPFLILTLTF
zfDisp     91   PGFCVCCSLC CNGNQQDCAL FQSAVPSAAL HMGSCCVQGV PCFCMQHHWP EHLQNQPNMA TLSPPRPFRF PKNYAELIAD WPVVVLGICT
                                                                  * .:           *   *   .  :.**.::*.  *.::* :

wbDisp     34   CANIVLLLVI VCTDSLPAFD DPQAGFEPRG SLINQRAQAW RNFLERDGVV QPSTRRKATT TKPVAARQNA PVQEIGTGNG DADYDFVEGL
zfDisp     181  VLIVVCALVG ILVPDLPDFS NPLQGFEPRG TAIGQRLVTW NNMVKNTGYK ATLANYPFKY AD----EQAK SHQ--DDRWS KDHYDRK---
                   :*  **  : . .** *. :*  ****** : *.**  :* .*:::. *       :.    .  :.     .*     *  .    . . .**

wbDisp     124  RNSTVKENVF SSSGMASLGP ITQPRGFFCS RPLLGHAKLI LRRKDAGNLL NLEGIRELCQ LDADIRRLDE FQSCSEESAD TGRLCRTWNL
zfDisp     262  --KRQ----- -------AEW DFSKDSFFCD VPGDSYSRIV FASAEGKNLW NIQAIKSMCN FDNTRVRSHP QYLDLCQRTT AASCCPSWTL
                  .                     .  .***.  *  .::::: :   :. **  *::.*:.:*: :*    * .           : :  :.  * :*.*

wbDisp     214  PNYVAVLAGK TSCHQLEEHD IRNVLNLTAT CLPFYRSGQL KADCSTSEDD GKTMQEDTIT CPGVPTDCIK DSSIYHLLYF ISDKESRETG
zfDisp     338  GNYIAVLTNK SSCQKITERD VSHTLNILRS CAKFYHNGTL GPECWDMTT- ----RKKDLK CGNLPRKCTK YNAVYQIFHF LVDKDFLNPK
                 **:***:.* :**::: *:* : :.**:  : *  **:.* *   :*                ::. :. * .:* .* *  .::*::::* : **:

wbDisp     304  ---YSSM--K HTLAVLPVWS SPAALPLYRA LQWGNF-TRP DDDGPIQVIG MEMGLGDRLF NTALLEDTLY VGVAAGVVVV VLWLYTGSLF
zfDisp     423  NTDYLPPNLK YSMLFAPTEK GETMMN---- IYLDNFENRN SSDGITTITG IEFGIKHSLF QDYLLTDTMY PAIAIVIVLV VMCVYTRSMF
                   *     *:::  . *. .. :  :        :  .** .* ..* *   : *:*: *: . ** :  ** **:* .: *  :*:**:  :** *:*

wbDisp     388  VTLMNLMAIF FSLVVSYFLY VFVFRLTFFP FMNLLTCVII VAIGADALVI FARLWHLAKT EKDDGRFEKV VHATFRHAAQ AILVSSLTAS
zfDisp     509  ITLMTMFAII SSLIVSYFLY RVVFNFDFFP FMNLTALIIL VGIGADDAFV LCDVWNYTKF DKPNSELSET VSITLQHAAL SMFVTSFTTA
                :***.::**:  **:****** .**.: *** **** : :*: *.****  .: :. :*: :*  :* :..:.:. *  *::***   :::*:*:*::

wbDisp     478  AALFSDIVNP IIAIRCFSIF AGLTVLVHLF FAVTWMPACF VVADKWGSSV YVCGVFAGTL LSC--DTKSP VCRLLYRCRC LHSGLRQYCD
zfDisp     599  AAFYANYVSN ITAIRCFGVY AGTAILVNYI LMVTWLPAVV VLHERYLVNL LTCSRTQNHQ SQGAATFWTS LCQV------ VNKCLFSISE
                **:::: *.  * *****.:: ** ::**: : : ***:** . *: :::  .:  .*.    .      .      :  :*::        ::. * . .:

wbDisp     566  YFRIFFEKLL PCLVIRLKWM WMASFVLLTV GACVVVFVFP GLQLASG--R TFSLWNSGHP SEQYRLLKDR FAFEENRNLN NNEKVSLHFV
zfDisp     683  ASRIFFEKVL PCVVIKLRYL WLLWFLALTV GGAYVVCVNP KMKLPSLELS EFQVFRSSHP FERYDAEYKK LFMFERVNHG EDLHMPITII
                  ******:* **:**:*::: *:   *: *** *.. ** * *   ::* *       *.::.*.**  *:*    .: : : *. * . :: :: : ::

wbDisp     654  WGVLALNQAA LLDPTDTGIT TMDGRFNMSD PLSQIWMLKF CADLRQQTFF DNS-TQSDNA CYFDSFMLWM ETGACSGH-- ------SQFP
zfDisp     773  WGVTPIDSGD PLNPKNKGKL TLDTTFNIAS PASQVWILNF CQKLRNQSFV YQSEEQDFTS CFMETFKQWM ENQDCEEASV YPCCSQSTFP
                ***  :::.   *:*.:.*   *:*  **::. * **:*:*:* * .**:*:*.  :*  *. .: *::::*  ** *.  *.            * **

wbDisp     735  YPPATFIRCV HTFSNSF--- ----PQAKHF GPNFNSLHQI DSFVLRLQTS QLFSHSYTAM QQLHQHVDQW FTAALRTAPP SLQGAWFTGD
zfDisp     863  YRQDVFELCI KRAIMELDRS TIYHLDSKTP GPRFDINDTI RAIVLEFQST YHFTLAYEKM HQFYREVDTW IQEELRDAPE GLSYGWFVSN
                *   .*  *: :     .:              ::*    **.*:   . *  ::**.:*::    *: :*   * :*:::.** * :    ** **   .*. .**..:

wbDisp     818  FAFFDLQQSL ISGTALSLIV SLFVAFLVLF FTTLNVGVSL IAITVIAGIM LATTAALVLM EWQLSVFEST IIGLAIGLSV DFTLHYAVSY
zfDisp     953  LEFYDLQDSL SDGTLIAMAL SVVVAFSVML LTTWNIIISL YAIISIAGTI FVTVGSLVLL GWELNVLESV TISVAVGLSV DFAVHYGVAY
                : *:***:**  .** ::: : *:.*** *:: :** *: :**  **  *** : :.*..:***:  *:*.*:**.  *.:*:**** **::**.*:*

wbDisp     908  CCAESYEERE LKTNIVISEM ASCVTMSAVT TFLAGALMIP SDILFYRQLG LFIITVTAIS LLYATIFLPA CLAVLGPQGA FLQFHYPSCR
zfDisp     1043 RLAPE-PDRE GKVVFSLSRM GSAIAMAALT TFVAGAMMMP STVLAYTQLG TFLMLIMCIS WAFATFFFQC MCRLLGPQGT CGQIPLPKKL
                  * .  :**  *. : :*.* .*.::*:*:* **:***:*:* * :* * ***  *:: : .**   :**:*: .     :*****:   *:  *.

wbDisp     998  PLCCRPDPSK LVEKSMHSSA EFDTTYTTGG TYDHHRETKQ CFHQSINQPY QGKALRSPSA AAN------- -NHHVVVISA TPEQPPKTHP
zfDisp     1132 --QCAERTSE NQ-------S TVNNQGKAGA CYTTKHEHYE LEPLAANVRN EGKASDEEQE ACTQLQNNIT SLHDDATLCA SHEEPKHEFE
                   *    *:            :  .:.  .:*.  *  ::*  :      : *    :***  . .  *..           *. ..:.* : *:* : .

wbDisp     1080 ALLLDV-DPD LA-------- ---DEETDSV GELG-SVPTS RGPSRGTSLI RRSQQASRGI FPWDYIMQRL PLSRHFSVAG SSVIYIDADV
zfDisp     1213 NGVQATEHSH LHQYAFNTRC TCENSMHQYI RERQRTACHP CGQSRETQCA TKQSNHLPVC LNRDM-IHAP TNSGHFQHCT PSQISRPAAH
                  :  . . . *                :.  : :  *   :.      * ** *.     :..:        :  *  ::      * **. .    * *   *

wbDisp     1156 RSVTSSMGL ---------- ---------- --------- ---------- ---------- ---------- ---------- ----------
zfDisp     1302 RCFSGGCGM HAVHISQQQT ETESYISACVP SSSPQESQN RPAHLQFPDV QRCSSPDTTG VCTLNHDTQN AGELAINSQS PQKIPWNHLKT
                *..:.. *:

wbDisp     1166 --EGPAVLPN RREDVPEVWV RRTTE----- ---------- ---------- ----------    ---------- ---
zfDisp     1392 KANGTCVLQD CREVVPEDSI GCRVDKEKTV SPKKFNCFKK NVNAKCNSVE FHKTRTSVPT    IAINSKASSE SLC
                  :* .** :  ** ***  :    .:
```

See next page for caption

Supplementary Fig. 3. Sequence homology between the water bear (*wb*) and zebrafish (*zf*) Hh and Disp proteins. Sequence alignment between the Hh (**A**) and Disp (**B**) of the two organisms. The **s**ignal peptide is shown in grey. An arrow marks the predicted cleavage site of the zebrafish Hh (*zf*Shh). ‘**-**’ indicates gap; ‘*’ indicates identical residues; ‘.’ and ‘:’ indicate weak and strong conservation, respectively.

```
wbDisp       ---------- ---------- ---------- ---------- ---------- ----------
ffDisp 1     MLCFDSERMN WYYHVLARRP YLVVVSIAVY CVACIIVALV LNKLPDFSDP TLGFETRGTK
hDisp        ---MAMSNGN NDFVVLSNSS IATSAANPSP LTPCDGDHAA QQLTPKEATR TKVSPNGCLQ

wbDisp 0     ---------- ---------- ---------- ---------- ---------- ----------
ffDisp 61    IG-------- ---------- ---------- ---------- ---------- -ERLTAWYNL
hDisp  58    LNGTVKSSFL PLDNQRMPQM LPQCCHPCPY HHPLTSHSSH QECHPEAGPA APSALASCCM

wbDisp 1     ---------- ---------- ---------- ---------- -------MPR QLVRRRKKTV
ffDisp 72    LQETDHHGAL FSNPSDLWER RRVEQGYVET KLHPNHRRRK NKHKNRNKNK RRKEQNQSSH
hDisp  118   QPHSEYSASL CPNHSPVYQT TCCLQPSPSF CLHHPWPDHF QHQPVQQHIA NIRPSRPFKL

                              TMH1
wbDisp 14    MQRYAHFLAH HPFLILTLTF CANIVLLLVI VCTDSLPAFD DPQAGFEPRG SLINQRAQAW
ffDisp 132   EHHDVAQKMM QFKKRLKATS SPSPNLGFDT WIGDSGVFRD YEITNDSASS SLEPTRRTEQ
hDisp  178   PKSYAALIAD WPVVVLGMCT MFIVVCALVG VLVPELPDFS DPLLGFEPRG TAIGQRLVTW
              :  .           *            :       .    .     . .. . :    *

wbDisp 74    RNFLERDGVV QPSTRRKATT TKPVAARQNA PVQEIGTGNG DADY----DF VEGLRNSTVK
ffDisp 192   IEYGHNTTSV DEEEHQQRVQ TKKSTWRLLK QAATLPTDGW ADMHRRQP-- ----------
hDisp  238   NNMVKN---- ---TGYKATL ANYPFKYADE QAKSHRDDRW SDDH----YE RE--------
              :  ..           : .  ::           .    .      :

wbDisp 130   ENVFSSSGMA SLGPITQPRG FFCS-RPLLG HAKLILRRKD A---GNLLNL EGIRELCQLD
ffDisp 240   ---------- -------IEG FFCDSSPRKE YSHFVVQRIG PNATDSLFDL NGLLAMCQLQ
hDisp  279   --------KR EVDWNFHKDS FFCD-VPSDR YSRVVFTSSG G---ETLWNL PAIKSMCNVD
                                 . ***.  *    :::.:.   .      .* :*  .:  :*:::

wbDisp 186   ADIRRLDEFQ SCSEESADTG RLCRTWNLPN YVAVLAGKTS CHQLEEHDIR NVLNLTATCL
ffDisp 283   DQITEVPSYR AFCEPEMLTT ECCRPWSLPN YAAMLANKSS CFDLTTEDVT SLHTLLLGCY
hDisp  327   NSRIRSHPQF GDLCQRTTAA SCCPSWTLGN YIAILNNRSS CQKIVERDVS HTLKLLRTCA
              .  .      .       :    * .*.* * * *:* .::* * .:  .*:     .*   *

wbDisp 246   PFYRSGQLKA DCSTSEDDGK TMQEDTITCP GVPTDCIKDS SIYHLLYFIS DKESRETGYS
ffDisp 343   EYFHDLKMDN HCN------- -----EIPHC RAPEECKRLN IVFNVLNFLT DFSFIKSNDS
hDisp  387   KHYQNGTLGP DCWDMAAR-- --RKDQLKCT NVPRKCTKYN AVYQILHYLV DKDFMTPKTA
              .::.  :   .*               :     .* .* : .  ::::* ::  * .   .  :

wbDisp 306   SMKHTLAVLP VWSSPAALPL YRALQWGNFT R--PDDDGPI QVIGMEMGLG DRLFNTALLE
ffDisp 390   NVYLKYAMIF IPVAQS---- NRLLPLFHEW EDVELINELV EVVAMDLGLE NELFNELLLT
hDisp  443   DYATPALKYS MLFSPTEKGE SMMNIYLDNF ENWNSSDGVT TITGIEFGIK HSLFQDYLLM
             .          :  : :            .   .     :     : .:::*:  . **:  **

                  TMH2 (SSD)                  TMH3 (SSD)                 TMH4
wbDisp 364   DTLYVGVAAG VVVVVLWLYT GSLFVTLMNL MAIFFSLVVS YFLYVFVFRL TFFPFMNLLT
ffDisp 446   DVWLVSLGGT FVMASVWLYT GSAFITLMSC VAICFSLGLA YFFYAIVLEF EFFPYMNLLA
hDisp  503   DTVYPAIAIV IVLLVMCVYT KSMFITLMTM FAIISSLIVS YFLYRVVFHF EFFPFMNLTA
             *.   .:.   .*:  : :**  * *:***.  .**  ** :: **:* .*:.:  ***:***  :

             TMH4 (SSD)                                                  TMH5
wbDisp 424   CVIIVAIGAD ALVIFARLWH LAKTEKDD-- ---------- ---------- GRFEKVVHAT
ffDisp 507   VVVIIGIGAD DVFLFLKIWH CVLTERFSNR CTLTTQSQSA LPTLENSDHT ESLENIMALT
hDisp  563   LIILVGIGAD DAFVLCDVWN YTKFDKPH-- ---------- ---------- AETSETVSIT
              ::::.****   .::  :*:  .  ::                              .: :  *

                  TMH5 (SSD)                               TMH6 (SSD)
wbDisp 462   FRHAAQAILV SSLTASAALF SDIVNPIIAI RCFSIFAGLT VLVHLFFAVT WMPACFVVAD
ffDisp 567   MRHAAASMFV TSLTTAGAFY ASYSSSITAI KCFGIFAGTV VVTNYLLMIT WLPASVSIME
hDisp  601   LQHAALSMFV TSFTTAAAFY ANYVSNITAI RCFGVYAGTA ILVNYVLMVT WLPAVVVLHE
             ::*** :::* :*:*::.*:: :.  . * ** :**.::** . ::.: .: :* *:** . : :
```

To be continued on the next page

Continued from the previous page

```
wbDisp   522   KWGSSVYVCG VFAGTLLSCD TKSPVCRLLY RCRCLHSGLR QYCDYFRIFF EKLLPCLVIR
ffDisp   627   RLFATRMSCH HPMS------ ---------- --IKLIHACK KSINRFCQMF EECITKSIMN
hDisp    661   RYLLNIFTCF KKP----QQQ IYDNKSCWTV ACQKCHKVLF AISEASRIFF EKVLPCIVIK
               :    .   *                                  :     :* *: :.   ::.

                   TMH7
wbDisp   582   LKWMWMASFV LLTVGACVVV FVFPGLQLAS GRT--FSLWN SGHPSEQYRL LKDRFAFEEN
ffDisp   669   YAYLWLLIFG ALGASSAVIV FWYPGLQLP- -EKSHFQLFV SKHPFEVYSS LKQQFWFEKP
hDisp    717   FRYLWLFWFL ALTVGGAYIV CINPKMKLPS LELSEFQVFR SSHPFERYDA EYKKLFMFER
                 ::*:  *   * .... :*    * ::*.   .   *.::  * ** * *     .:: : :

wbDisp   640   RNLNNNEKVS LHFVWGVLAL NQAALLDPTD TGITTMDGRF NMSDPLSQIW MLKFCADLRQ
ffDisp   727   LQAYENFKMH MHFVWGVQAV DDGDYTNPNS YGHLHYDNNF NVSSRPAQLW ILDFCQSVRQ
hDisp    777   VHHGEELHMP ITVIWGVSPE DNGNPLNPKS KGKLTLDSSF NIASPASQAW ILHFCQKLRN
                :  :: ::  : .:*** .  ::.   :*..  *      *. * *::.  :* * :*.** .:*:

wbDisp   700   QTFFDNSTQS DN-ACYFDSF MLWMETG--- ---------A CSGHSQFPYP PATFIRCVHT
ffDisp   787   QPFYKETLGM LLPNCFIENL IDYMKRRCID DMDSTRKDRS PCCDAQFPFE PHIFEYCLPQ
hDisp    837   QTFFYQTDEQ DFTSCFIETF KQWMENQDCD EPALY----P CCSHWSFPYK QEIFELCIKR
               *.*: ::         *:::.:   :*:                .  . . .**:       *  *:

wbDisp   747   FSNSFP---- ---------- ---------- --------QA KHFGPNFNSL HQIDSFVLRL
ffDisp   847   SISNMYDTTF FRPGVAGPKF AEAPRLETED YLGMSGNESA EYSTNGSFTP LLVKALVIEF
hDisp    893   AIMELERSTG YHL------- ---------- --------DS KTPGPRFDIN DTIRAVVLEF
                  .:                                   .: :             : :.*:.:

wbDisp   775   QTSQLFSHSY TAMQQLHQHV DQWFTAALRT APPSLQGAWF TGDFAFFDLQ QSLISGTALS
ffDisp   907   ESNVAYSTIY ANIRQFYESV EHWFQMQLKT APPELQGGWF TSDLKFYNVQ DTLSHDTFVA
hDisp    928   QSTYLFTLAY EKMHQFYKEV DSWISSELSS APEGLSNGWF VSNLEFYDLQ DSLSDGTLIA
               ::.  ::  *    ::*::: * : *:    * : **  *...** ..:: *:::* ::*   .* ::

                 TMH8                        TMH9                          TMH10
wbDisp   838   LIVSLFVAFL VLFFTTLNVG VSLIAITVIA GIMLATTAAL VLMEWQLSVF ESTIIGLAIG
ffDisp   966   ICLAMAASLA VLLCFTVNIL ISIYAVLTVS LSIFNTVAVL ILLGWQLNIL ESIAVSTAIG
hDisp    988   MGLSVAVAFS VMLLTTWNII ISLYAIISIA GTIFVTVGSL VLLGWELNVL ESVTISVAVG
               : ::: .::  *::  * *:  :*: *:  ::   :: *.. * :*: *:*.:: **   :. *:*

                 TMH10                                 TMH11
wbDisp   895   LSVDFTLHYA VSYCCAESYE ERELKTNIVI SEMASCVTMS AVTTFLAGAL MIPSDILFYR
ffDisp   1027  LAVDFSLHYG IHYRMS-PVK ERLAATQFVL SRIIGPTVMA ATTTGLAGGI MMASNILPYI
hDisp    1048  LSVDFAVHYG VAYRLAPDPD -REGKVIFSL SRVGSAMAMA ALTTFVAGAM MMPSTVLAYT
               *:***::**. : *   :    .   *    . : : *.: .   .*: * ** :**.: *:.* :* *

                      TMH12
wbDisp   955   QLGLFIITVT AISLLYATIF LPACLAVLGP QGAFLQFHYP SCRPLCCR-- ----------
ffDisp   1086  QIGVFLVVVM IVSWFYATFF LMSLLRVAGP QHGFLELKWP LWSKRSSG-- ----------
hDisp    1107  QLGTFMMLIM CISWAFATFF FQCMCRCLGP QGTCGQIPLP KKLQCSAFSH ALSTSPSDKG
               *:* *:: :   :*   :**:* : .        ** *     ::  *          ..

wbDisp   1003  ---------- -PDPSKLVEK SMHSSAEFDT TYTTGGTYDH HRETKQCFHQ SINQPYQGKA
ffDisp   1134  ---------- ---SSKFYER KPSQVIASEQ LLTPTSSAIV ELANSETHEL ESLN------
hDisp    1167  QSKTHTINAY HLDPRGPKSE LEHEFYELEP LASHSCTAPE KTTYEETHIC SEFFNSQAKN
                               .     ..     .      :     :    :    .   .: .   .

wbDisp   1052  LRSPSAAANN ---------- ---------- ---------- ---------- ----------
ffDisp         ---------- ---------- ---------- ---------- ---------- ----------
hDisp    1227  LGMPVHAAYN SELSKSTESD AGSALLQPPL EQHTVCHFFS LNQRCSCPDA YKHLNYGPHS
```

To be continued on the next page

Continued from the previous page

```
wbDisp  1052  LRSPSAAANN ---------- ---------- ---------- ---------- ----------
ffDisp        ---------- ---------- ---------- ---------- ---------- ----------
hDisp   1227  LGMPVHAAYN SELSKSTESD AGSALLQPPL EQHTVCHFFS LNQRCSCPDA YKHLNYGPHS

wbDisp  1062  ---------- ---------- ---------- ---------- HHVVVISATP EQPPKTHPAL
ffDisp        ---------- ---------- ---------- ---------- ---------- ----------
hDisp   1287  CQQMGDCLCH QCSPTTSSFV QIQNGVAPLK ATHQAVEGFV HPITHIHHCP CLQGRVKPAG

wbDisp  1082  LLDVDP---- ---------- ---------- ---------- ---------- --------DL
ffDisp        ---------- ---------- ---------- ---------- ---------- ----------
hDisp   1347  MQNSLPRNFF LHPVQHIQAQ EKIGKTNVHS LQRSIEEHLP KMAEPSSFVC RSTGSLLKTC

wbDisp  1090  ADEETDSVGE LGSVPTSRGP SRGTSLIRRS QQASRGIFPW DYIMQRLPLS RHFSVAGSSV
ffDisp  1175  ---------- ------SNSL IKTISGIESA HALSSLPRDF EHSFQTMHEC KYQTYPSTSN
hDisp   1407  CDPENKQREL CKNRDVSNLE SSGGTENKAG GKVELSLSQT DASVNSEHFN QNEPKVLFNH
                               *.       :  . .    .       :  .:      :  .    .

wbDisp  1150  IYIDADVRSV TSSMGLEG-- --------PA VLPNRREDVP EVWVRRTTE- --------
ffDisp        ---------- ---------- ---------- ---------- ---------- --------
hDisp   1467  LMGEAGCRSC PNNSQSCGRI VRVKCNSVDC QMPNMEANVP AVLTHSELSG ESLLIKTL
```

Supplementary Fig. 4. Sequence alignment of Disp homologs from human, water bear, and fruit fly. Symbols: '-' indicates gap; '*' indicates identical residues; '.' and ':' indicates weak and strong conservation, respectively. For secondary structures, line indicates random coil, cylinder marks α-helix, and arrow indicates β-strand. Transmembrane helices (TMH) are colored red. Only the secondary structures of the *wb*Disp are labeled. The sterol sensing domain (SSD) contained in the THM2-5 shows higher homology than the rest part of the protein. Sequences marked in green indicate deleted residues in the construct used for structure determination.

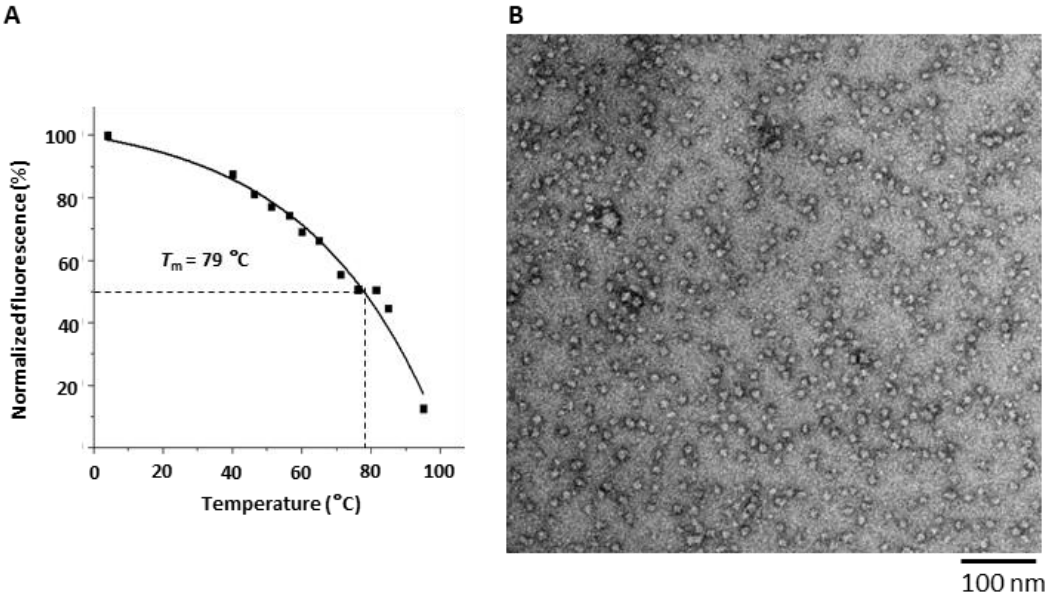

Supplementary Fig. 5. Characterization of the optimized construct of water bear Disp. (**A**) Pseudomelting curve of the protein. The protein fused with a fluorescence reporter was heated at indicated temperatures before being analyzed by fluorescence-detection size exclusion chromatography. The peak intensity is plotted as a function of heating temperature and the apparent melting temperature (T_m) was obtained by regression fitting. (**B**) Negative-staining of the protein purified in digitonin.

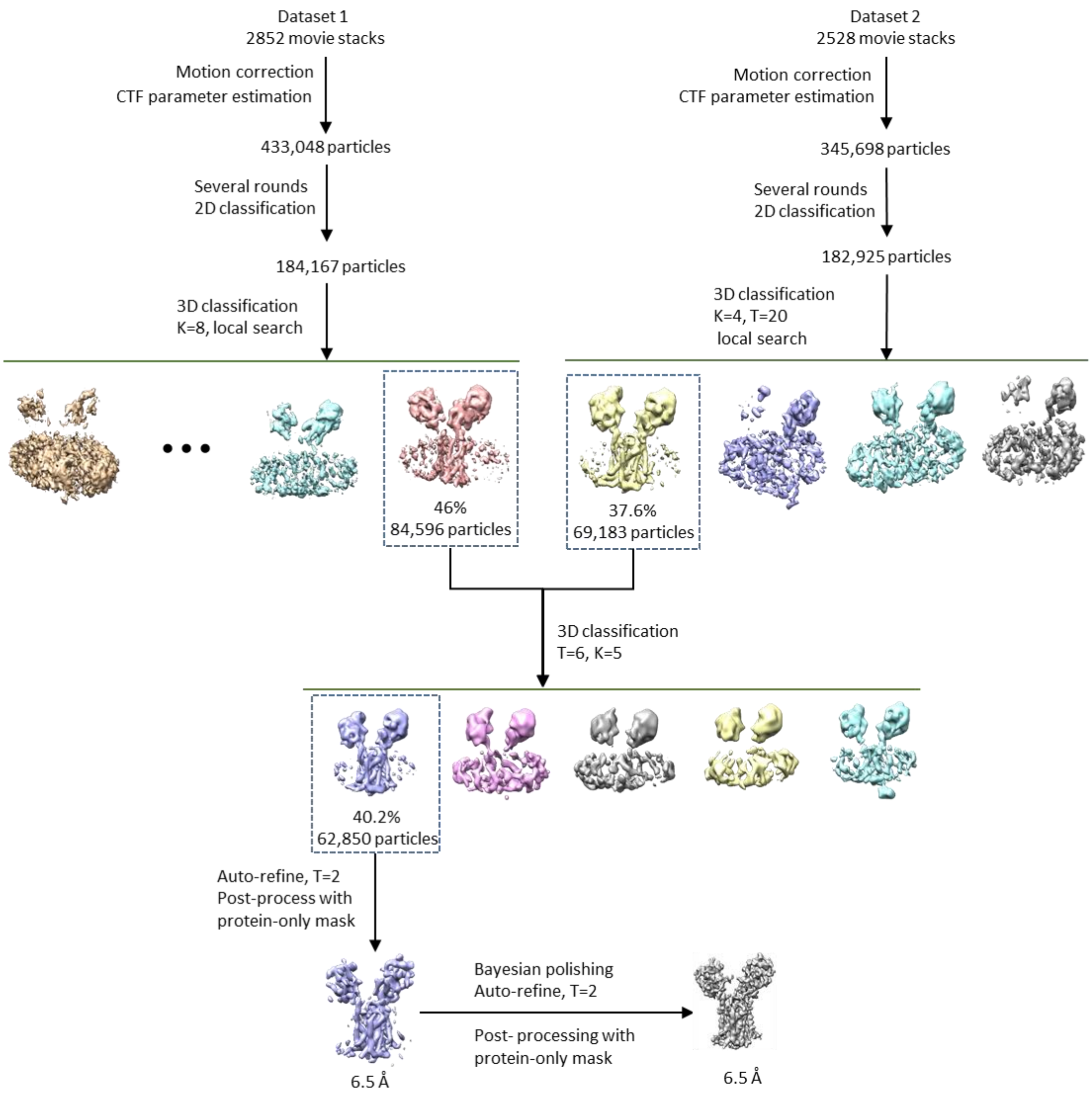

Supplementary Fig 6. Data processing flowchart. T is the regularization parameter and K is the number of classes during classification.

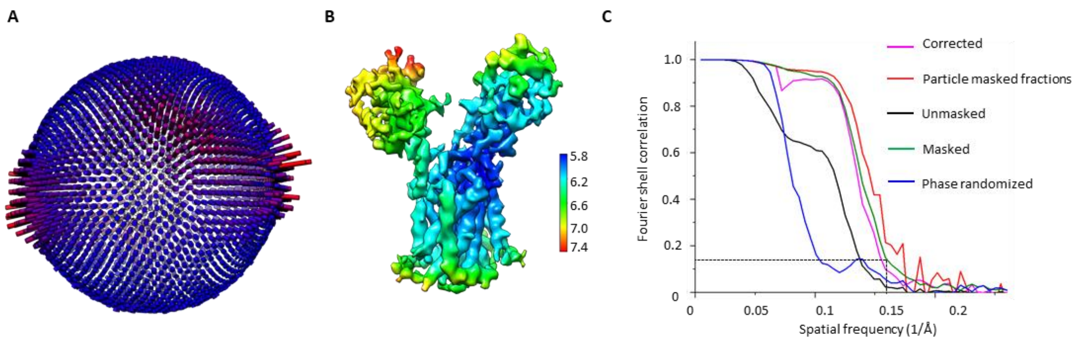

Supplementary Fig. 7. Quality of cryo-EM data and map. (**A**) Angluar distribution of *wbDisp* generated by Relion (14, 15). Each cylinder represents one view, and the height is scaled to the number of particles in that view. (**B**) Local resolution of the final density map estimated by Relion. (**C**) Gold-standard Fourier shell correlation (FSC) curves of the final density maps (generated by Relion).

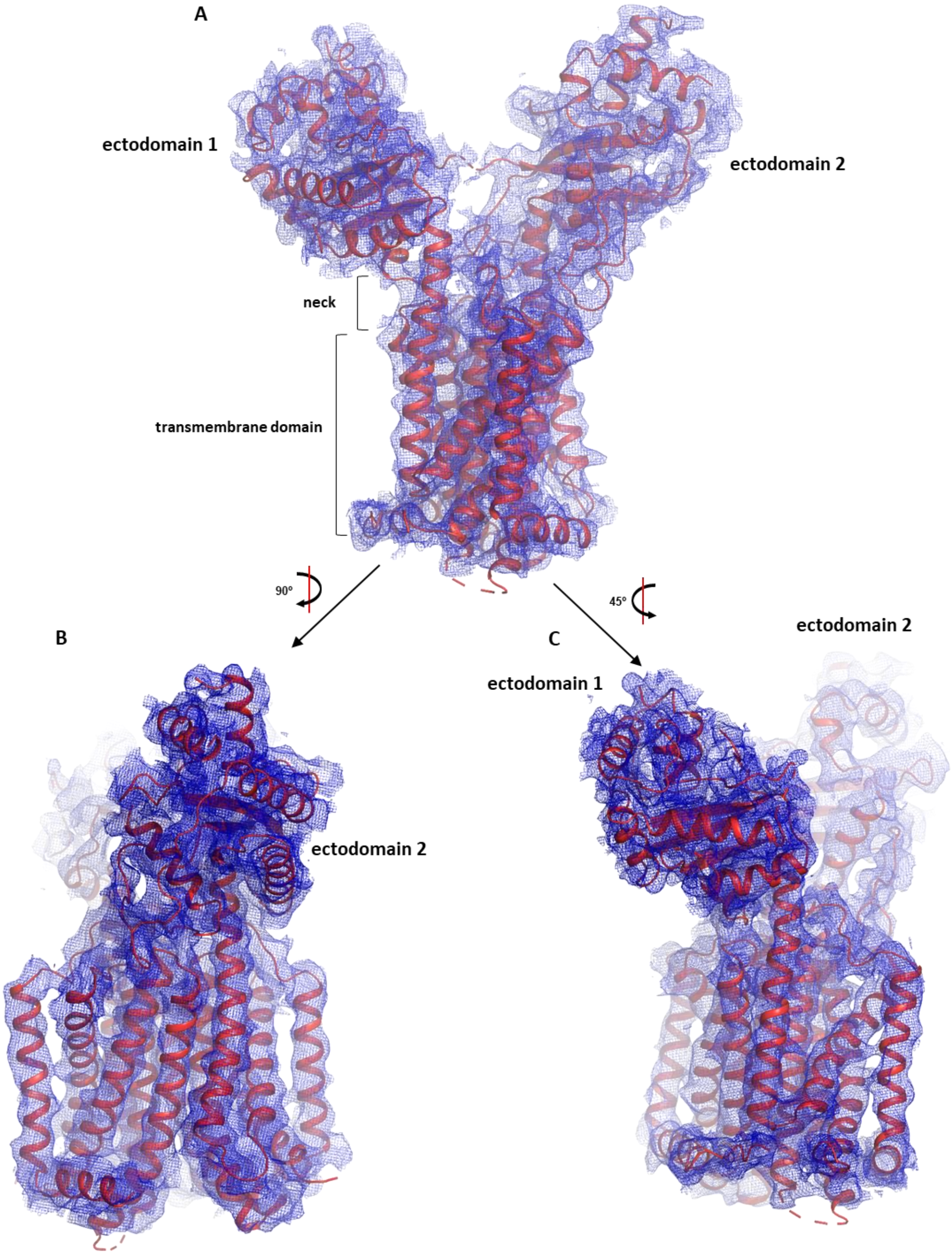

Supplementary Fig. 8. Cryo-EM density map of *wb*Disp. (**A-C**) Different views of *wb*Disp (red) and the cryo-EM density map (blue mesh).

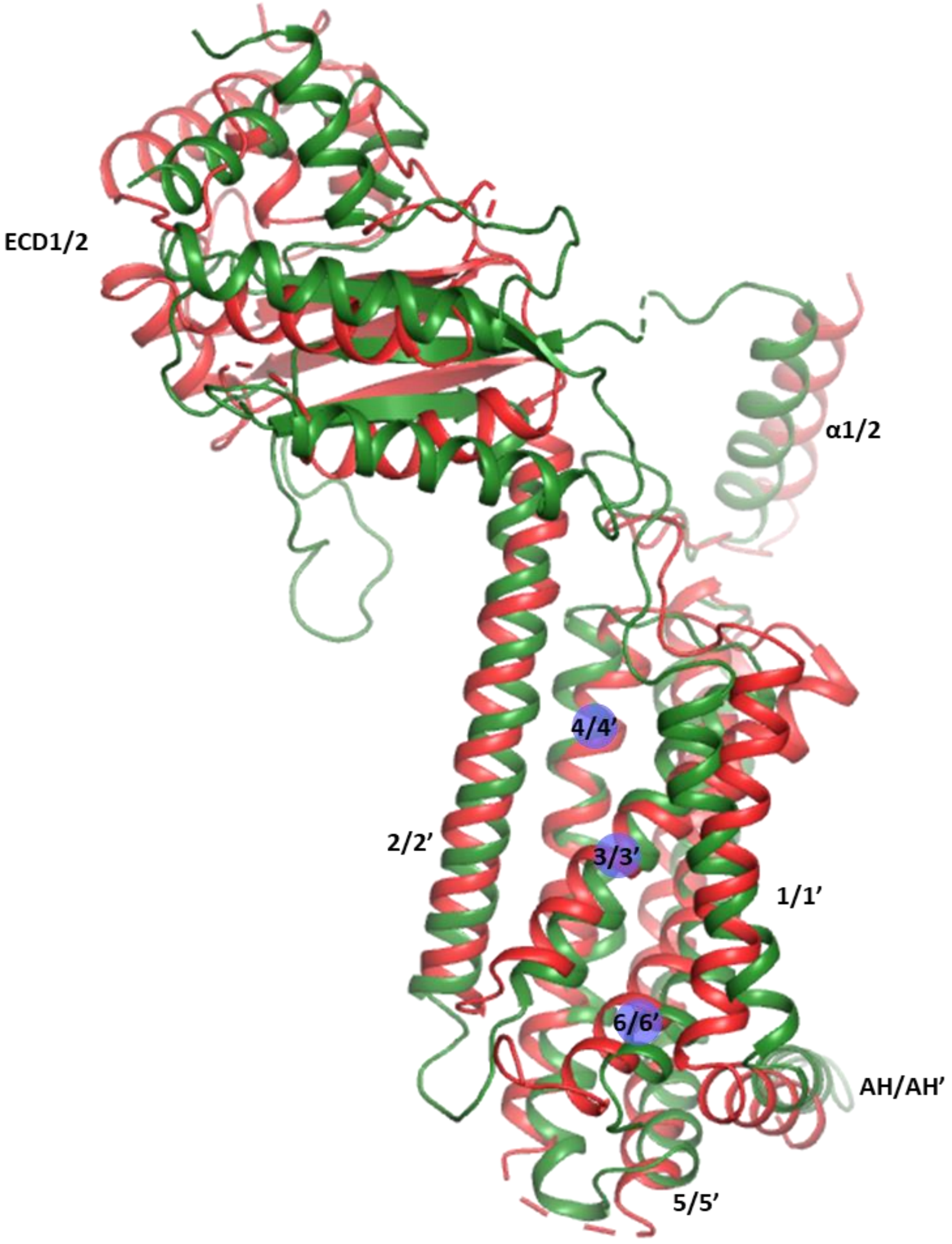

Supplementary Fig. 9. *wb*Disp displays pseudosymmetry between the N-terminal (red) and C-terminal half (green). Various parts as in Fig. 1D are labeled. The two parts, when aligned, have a Cα root mean square deviation of 5.059 Å.

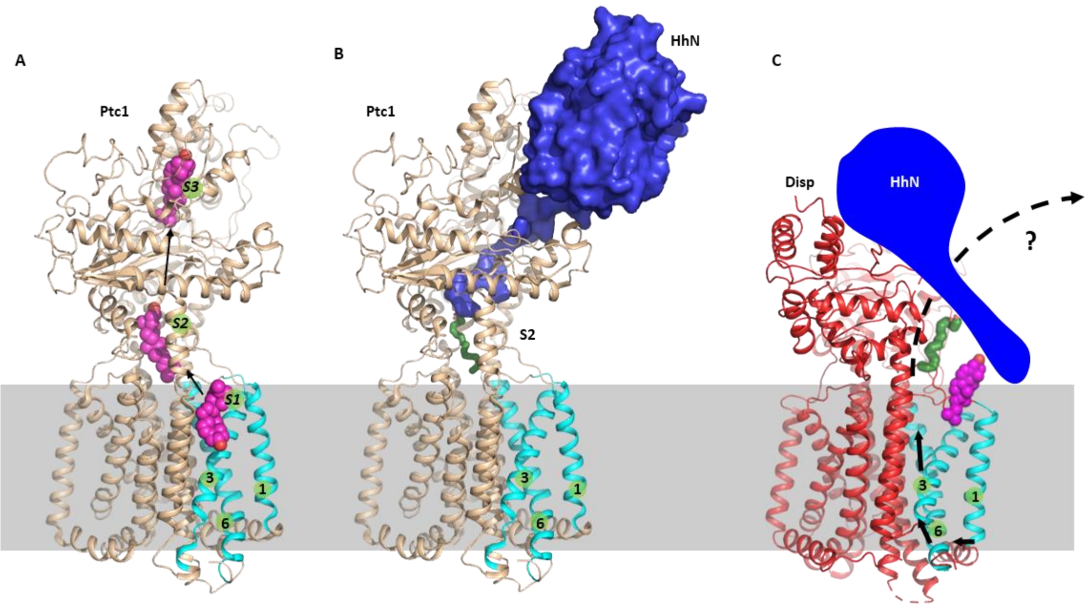

Supplementary Fig. 10. The conformational changes in the transmembrane domain may be related to Disp's function. (**A**) The structure of Ptc1 (PDB ID 6mg8) (12). Sterol molecules in the three sites (*S1*-*S3*) along the sterol conduit are shown as magenta spheres. Black arrows show the direction of cholesterol transport. The sterols are taken from the PDB 6mg8 (12); the *S1* sterol may assume a flipped conformation with its 3-OH group at the membrane interface. (**B**) The Ptc1 structure (wheat and cyan) in complex with HhN (blue) (PDB ID 6oev) (16). The palmitoyl chain occupying Site 2 (*S2*) of the sterol conduit is shown as green sticks. In **A** and **B**, the transmembrane helices (TMH 1, 3, and 6) corresponding to *wb*Disp TMHs that show conformational differences are colored cyan. (**C**) The TMH movement may be related to Hh release. The ligand HhN, drawn as N-terminally palmitoylated (green) and C-terminally cholesterylated (magenta), is schematically placed for illustration purposes. The ligand is not drawn in scale. Straight arrows indicate the conformation change of the TMHs (cyan) which may facilitate the release of the palmitoyl and/or the cholesteryl chain from the membrane. A curved arrow indicates the release of HhN. Numbers label TMH 1, 3, and 6.

Supplementary Table 1. Data collection and refinement statistics.

	*wb*Disp*
**Data collection and processing**	
Magnification	22,500
Voltage (kV)	300
Electron exposure (e$^-$ Å^{-2})	60.8
Defocus range (μm)	-1.5 to -2.5
Pixel size (Å)	1.0
Initial particles (no.)	778,746
Final particles (no.)	62,850
Map resolution (Å)	6.5
FSC threshold	0.143
Map sharpening B factor (Å^2)	-365.64
**Refinement**	
Model composition	
Protein residues	805
Nonhydrogen atoms	4079
B-factors (Å^2)	173.7
Validation	
MolProbity score	1.71
Clash score	5.58
CC mask	0.76
CC box	0.53
CC volume	0.76
CC peaks	0.37
CC main chain	0.66
R.m.s deviations	
Bond lengths (Å)	0.006
Bond angles (°)	0.994
Ramachandran	
Favoured (%)	93.9
Allowed (%)	6.1
Outlier (%)	0.0
**PDB ID**	7FIF

Supplementary References

1. Porter JA, Young KE, Beachy PA. Cholesterol modification of hedgehog signaling proteins in animal development (1996). *Science* **274** (5285): 255-259.
2. Porter JA, Ekker SC, Park WJ, von Kessler DP, Young KE, Chen CH, et al. Hedgehog patterning activity: role of a lipophilic modification mediated by the carboxy-terminal autoprocessing domain (1996). Cell **86** (1): 21-34.
3. Pepinsky RB, Zeng C, Wen D, Rayhorn P, Baker DP, Williams KP, et al. Identification of a palmitic acid-modified form of human Sonic hedgehog (1998). *J Biol Chem* 273 (22): 14037-14045.
4. Mao H, Diehl AM, Li YX. Sonic hedgehog ligand partners with caveolin-1 for intracellular transport (2009). *Lab Invest* **89** (3): 290-300.
5. Hall ET, Dillard ME, Stewart DP, Zhang Y, Wagner B, Levine RM, et al. Cytoneme delivery of Sonic Hedgehog from ligand-producing cells requires Myosin 10 and a Dispatched-BOC/CDON co-receptor complex (2021). *eLife* **10**: 61432.
6. Creanga A, Glenn TD, Mann RK, Saunders AM, Talbot WS, Beachy PA. Scube/You activity mediates release of dually lipid-modified Hedgehog signal in soluble form (2012). *Genes Dev* **26** (12): 1312-1325.
7. Tukachinsky H, Kuzmickas RP, Jao CY, Liu J, Salic A. Dispatched and scube mediate the efficient secretion of the cholesterol-modified hedgehog ligand (2012). *Cell Rep* **2** (2): 308-320.
8. Koleva MV, Rothery S, Spitaler M, Neil MA, Magee AI. Sonic hedgehog multimerization: a self-organizing event driven by post-translational modifications? (2015) *Mol Membr Biol* **32** (3):65-74.
9. Simon E, Aguirre-Tamaral A, Aguilar G, Guerrero I. Perspectives on Intra- and Intercellular Trafficking of Hedgehog for Tissue Patterning (2016). *J Dev Biol* **4** (4): 34.
10. Panakova D, Sprong H, Marois E, Thiele C, Eaton S. Lipoprotein particles are required for Hedgehog and Wingless signalling (2005). *Nature* **435** (7038): 58-65.
11. Eugster C, Panakova D, Mahmoud A, Eaton S. Lipoprotein-heparan sulfate interactions in the Hh pathway (2007). *Dev Cell* **13** (1): 57-71.
12. Zhang Y, Bulkley DP, Xin Y, Roberts KJ, Asarnow DE, Sharma A, et al. Structural Basis for Cholesterol Transport-like Activity of the Hedgehog Receptor Patched (2018). *Cell* **175** (5): 1352-1364.
13. Niewiadomski P, Niedziolka SM, Markiewicz L, Uspienski T, Baran B, Chojnowska K. Gli Proteins: Regulation in Development and Cancer (2019). Cells **8** (2): 147.
14. Scheres SH. A Bayesian view on cryo-EM structure determination (2012). *J Mol Biol* **415** (2): 406-418.
15. Scheres SH. RELION: implementation of a Bayesian approach to cryo-EM structure determination (2012). *J Struct Biol* **180** (3): 519-530.
16. Qi X, Schmiege P, Coutavas E, Wang J, Li X. Structures of human Patched and its complex with native palmitoylated sonic hedgehog (2018). *Nature* **560** (7716): 128-132.
